# The third wave of biological psychiatry

**DOI:** 10.3389/fpsyg.2013.00582

**Published:** 2013-09-05

**Authors:** Henrik Walter

**Affiliations:** ^1^Research Division of Mind and Brain, Department of Psychiatry and Psychotherapy, Charité Universitaetsmedizin BerlinBerlin, Germany; ^2^Berlin School of Mind and Brain, Humboldt UniversityBerlin, Germany

**Keywords:** mental disorder, cognitive neuroscience, neuroimaging, genetics, philosophy of mind, philosophy of psychiatry, RDOC, DSM-5

## Abstract

In this article I will argue that we are witnessing at this moment the third wave of biological psychiatry. This framework conceptualizes mental disorders as brain disorders of a special kind that requires a multilevel approach ranging from genes to psychosocial mechanisms. In contrast to earlier biological psychiatry approaches, the mental plays a more prominent role in the third wave. This will become apparent by discussing the recent controversy evolving around the recently published DSM-5 and the competing transdiagnostic Research Domain Criteria approach of the National Institute of Mental Health that is build on concepts of cognitive neuroscience. A look at current conceptualizations in biological psychiatry as well as at some discussions in current philosophy of mind on situated cognition, reveals that the thesis, that mental brain disorders are brain disorders has to be qualified with respect to how mental states are constituted and with respect to multilevel explanations of which factors contribute to stable patterns of psychopathological signs and symptoms.

## WHAT IS BIOLOGICAL PSYCHIATRY?

As a first approximation we can say that it ties psychiatry closely to the biology of the brain. Under such a broad characterization today nearly everyone would qualify as a biological psychiatrist, as only very few would deny such a connection. However, there are stronger and more controversial claims, for example the ontological claim that psychiatric disorders are disorders of the brain, or, on the therapeutic level, that the best therapies are biological ones like medication or deep brain stimulation. However, many biological psychiatrists would not share these stronger claims, so this characterization seems too narrow.

To better understand the characteristics of the third wave, it will be helpful to take a short look at the first and second wave in the history of psychiatry (Shorter, 1998). The first wave in the second half of the nineteenth century can be best understood as a new research agenda. It was not so much characterized by the idea that the mental and the nervous system are closely linked – this was already believed by ancient philosophers – but rather by the ambition to uncover the relation between mind and brain by doing systematic research linking neuropathology and mental disorder and by using the experimental method in animals and humans. Wilhelm Griesinger (1817–1868), one of the most important figures of this first wave, famously declared: mental disorders are disorders of the brain. Note, that this was not primarily intended as a reductionist claim, but rather as a statement intended to delineate his ideas against the two prevailing approaches of that time: the moral approach on the one hand, and the somatic approach, linking mental disorder to body processes in the lung, liver or other organs, on the other hand. Nevertheless, Griesingers claim was not at all uncontroversial as theorists felt that such a brain approach would not do justice to the intricate psychopathological phenomena psychiatrists dealt with. For example, Karl Jaspers, the philosopher-psychiatrist, called 1913 the localationist models of two main protagonists of the first wave, Theodor Meynert and Carl Wernicke, “brain mythologies.”

In the early twentieth century, there was a decline in the biological approaches through various developments. Emil Kraeplin, one of the most influential psychiatrists at his time, started as an opponent to biological psychiatry, and developed his diagnostic system on systematic observations of symptoms and course of mental disorders, laying the groundwork for the later DSM. Also, psychological models, inspired by psychoanalysis and behaviorism became increasingly fashionable and had a large impact on therapy.

The second wave of biological psychiatry started only in the second half of the twentieth century and was, according to Shorter, driven by two new discoveries. The first was genetics, which could show that severe mental disorders, in particular schizophrenia, have a strong genetic component. The second was the discovery of efficient medication for various mental disorders (1949 lithium, 1952 chlorpromazin, 1957 imipramin, 1958 haloperidol, 1963 diazepam). They quickly became a major pillar of psychiatric treatment and contributed strongly to the opening and later disappearance of the large mental asylums in the second half of the last century. Soon, the concept of a neurochemical imbalance of neurotransmitters became the favored explanatory model for psychiatric disorders. Interestingly, at the same time as psychiatry for the first time used effective medications, the movement of antipsychiatry emerged. It was part of a more general political protest against tradition starting in the 1960s and declared “mental illness as a myth” (Szasz, 1961). It also was quite effective in discrediting one of the most effective treatments for severe depression, electroconvulsive therapy, supported among other things by the impressive movie “One flew over the cockoo’s nest” (1975) by Milos Forman. So although the second wave was in effect quite successful there was always some opposition against it on one hand, but on the other hand those insights and practices that were helpful for patients are now integrated into daily practice.

So what is the third wave of biological psychiatry? I want to suggest that this wave has started in the last two decades of the twentieth century and is now in full progress. Again, it has been driven by methodological and technological progress. Since the declaration of the last decade of the twentieth century as the decade of the brain by the president of the United States, neuroscience has developed into one of the largest research programs worldwide. According to my view, there were two developments particularly relevant in the transition of the second wave into the third wave. The first is the progress in the molecular neurosciences. The journal *Molecular Psychiatry*, founded in 1997, is now one of the fields most prestigious and most cited journals. It became increasingly clear that the effects of psychiatric drugs are not primarily exerted via the level of neurotransmitters in the synaptic cleft, but that there is up- and down-regulation of receptors, effects on intracellular cascades, and even regrowth of neurons in the hippocampus. The picture of the neurobiological changes underlying psychiatric disorders and treatment thus became much more complex and differentiated and it became apparent that different levels of brain organization are important which interact in a complex way. The second development was the birth of cognitive neuroscience and neuroimaging. This field studies information processing in the brain by combining the methods of experimental psychology with tools to record brain activity or to stimulate the brain. In fact, neuroimaging, in particular functional magnetic resonance imaging (fMRI) has contributed much to public “brain awareness,” by (although wrongly) suggesting that we can literally watch “the brain at work.” With the first human study published in 1991, fMRI has today become a major research tool in psychology as well as in psychiatry. This development could not have taken place without a large increase in computational power. In fact, computational neuroscience which tries to develop mathematical models of brain function, has become an important tool in explaining neurocognitive processes and recently the program of computational psychiatry has begun to evolve (Montague et al., 2012). Further methods and technologies have become available to investigate the interplay of genetics, experience and environment in the etiology and neural explanation of psychiatric disorders like imaging genetics, epigenetics, optogenetics, or deep brain stimulation. Also big science, combining large – omic datasets like the (epi)genom, metabolom, proteome, or connectom with clinical data is becoming more important in psychiatric research and allows for new ways of discovery. The underlying model is that of systems medicine, understood as an interdisciplinary field of study that looks at the dynamic systems of the human body as part of an integrated whole, incorporating biochemical, physiological, and environmental interactions that sustain organismic life. In brain science, the paradigm of localationist thinking is substituted increasingly by thinking in functional systems and brain connectivity patterns (Buckholtz and Meyer-Lindenberg, 2012).

At this moment, we are at a critical stage of the third wave. In fact, progress in the first decade of this century has been so impressive that researchers as well as media have been overenthusiastic with regard to the power of the new methods. In particular neuroimaging results, probably due to their seemingly simple and straightforward presentation, have ignited the imagination of researchers, lay people and the media. Results are reported, similar to genetic results, in a oversimplified causal language (“love is in the ACC,” “the God spot,” “gene for schizophrenia discovered,” etc.). Such oversimplified messages are well for drawing attention to headlines, but way over what really can be inferred from most studies. Consequently, neuroscience has recently been criticized for its overambitious claims, and the field of “critical neuroscience” has flourished in the last 5 years immensely with an increasing number of books, papers and blogs (for a respectable example compare Slaby and Choudhury, 2011). Actually, in neuroscience in general, as well as in cognitive neurosciences and neuroimaging in particular self-critical articles concerning methods have begun to be increasingly published (e.g., Kriegeskorte et al., 2009; Button et al., 2012) which is a healthy self-correcting development.

According to the third wave of biological psychiatry, mental disorders are relatively stable prototypical, dysfunctional patterns of experience and behavior that can be explained by dysfunctional neural systems at various levels. As with any understanding of disease in general the notion of a “dysfunction” inevitable involves normative judgments of what is regarded as normal, functional, healthy on the one hand, and as abnormal, dysfunctional, pathological on the other hand. Further below I will come back to normative issues. But before I do so, let’s look at the concept of mental disorder within biological psychiatry.

## WHAT ARE MENTAL DISORDERS?

Modern psychiatry has taken a lot of effort to make the description of psychopathology reliable by introducing standardized ways of exploring, describing and rating psychopathological patterns over time. In America, psychiatric disorders are diagnosed using the DSM-IV (published 1994), the Diagnostic Statistics and Manuals of Mental Disorders, the official handbook of the American Psychiatric Association (APA), sometimes referred to as the “bible” of psychiatry. According to DSM-IV mental disorders are diagnosed by carefully checking if subjects fulfill a certain number of psychopathological criteria for a certain amount of time. DSM-IV is agnostic on etiopathogenesis, i.e., the causal genesis of disorders, but rather has put emphasis of establishing a reliable, intersubjective schema for diagnoses on the psychopathological level. But what about validity, i.e., what is measured or rather intended to be measured with DSM-criteria? What kind of things are mental disorders? Kendler et al. (2011a) have distinguished four types of kinds that mental disorders could be. *Essentialist kinds *are based on an essence, e.g., an underlying cause, from which the defining features (the typical symptoms) do arise. Although this theory fits to some cases like progressive paralysis in syphilis or Mendelian defects in cholesterol metabolisms, it is now widely acknowledged that this model neither fits most chronic diseases as atherosclerosis, hypertension or autoimmune disease, nor psychiatric disorders. Rather, it is generally accepted that psychiatric disorders arise from a multitude of causes that are probabilistically related to signs and symptoms. Even in cases, where family and twin studies unambiguously have demonstrated that most of the variance is explained by genetics factors (e.g., up to 80% in schizophrenia) there is no single gene causing this disorder. Recently discovered risk variants explain only a tiny portion of variance, usually less than 1%, although, using imaging genetics, they can be shown to have much stronger effects on the brain level (Walter et al., 2011). A second approach is to understand psychiatric disorders as *socially constructed kinds *which are brought about solely by the human activity of describing and classifying but not by an underlying structure independent of human constructs. Although this still is a popular thesis in the camps of cultural relativists and anti-psychiatry, this theory is rarely taken serious today. Instead, it is now widely acknowledged that cultural influences and social factors play important roles in the expression of symptoms, e.g., in the content of delusions. But it is also clear that for certain prototypical diseases (e.g., schizophrenia, bipolar disorder, depression, and some anxiety disorders) there are invariant patterns in experience and behavior despite eminent cultural differences. Many people think that what matters most is how we handle mental problems. So maybe psychiatric disorders are best understood as *practical kinds*. This approach holds that psychiatric disorders do not carve nature at its joints but just are those kinds which are most useful for certain purposes, ranging from medical ones (diagnoses and treatment) to sociological or even political ones (this is the point of departure of anti-psychiatry). This model is grounded in pragmatist philosophy and instrumentalism and has some appeal. In fact, the philosophy of DSM is very close to this approach with its agnostic and atheoretical framework. Although the practical kind of view avoids metaphysical discussion (like: What is schizophrenia really?), it gives us no advice as how classifications should be build and goes against many realistic intuitions that are the basis of successful applications not only in medicine. Instead, Kendler et al. (2011a) argue for a concept that is based on a model originating in the philosophy of biology, dealing with the problem how species are classified and on recent developments in the theory of neuroscience: the *concept of mechanistic property clusters (MPC)*. According to this view, the items to be classified rest on properties that need not to be shared by all members of a class, rather they should be understood as a cluster within an abstract space of features or properties in a multidimensional space. Some of those features may be more essence like, some more practical. Importantly, the MPC-view encourages the thought that there are robust explanatory structures to be discovered underlying psychiatric disorders. These explanatory multidimensional structures (genes, cell receptors, neural systems, psychological states, environmental inputs, social-cultural variables) are interacting in a complex and intertwined way, are sometimes fuzzy, but nevertheless stable. Importantly, it cannot simply be read from the superficial structure of items if they belong to the same kind. Rather, their membership is explained by the causal mechanisms that regularly ensure that their properties are instantiated together (a historical account). The interaction typically is inter-level, but can also be on the level on symptoms, thus mutually re-enforcing the pattern, e.g., in depression insomnia predisposes to tiredness and guilt predisposes to suicidal ideation. As MPC kinds are defined in part by the mechanisms that underlie and sustain them, the reductionist intuitions of old wave biological psychiatry are partially satisfied. However, the kind cannot be fully explained and thus understood if inter-level interactions, which are often hidden to the subject as well as to the external observer, are not taken into account. For example, it has been empirically shown that subjective explanations for depressive episodes by patients do not correlate with objective risk factors for depression (Kendler et al., 2011b) – a finding that makes it likely that explanations based on just a selection of levels (subjective experience and remembered behavioral events) do not explain depression well. The same can be said for simplified biological models of depression as a neurotransmitter deficit that ignores many of the other levels. Although the MPC-model does not tell us in advance what the relevant causal mechanisms are, it is consistent with the new biological wave in psychiatry which we will now characterize by describing a controversy around the introduction of DSM-5.

## DSM-5 AND ITS CRITICS

On May 18th 2013 DSM-5 was launched at the meeting of the APA. When the APA started to work on DSM-5, it was hoped that it would be able to integrate new dimensional approaches (constellation of symptom dimensions, rather than categories of disorders) and more of the exploding neurobiological research results from the molecular and cognitive neurosciences. However, this hope was frustrated. Shortly before publication, the APA-DSM task force decided against these ideas, as it felt it would be too early and that research was not far enough to deliver sound evidence that could be integrated. Moreover, another feature of DSM-5 steered much controversies. Diagnostic criteria for some disorders were changed and new disorders were included. For example, the former exclusion criterion for the minimum duration of a depressive episode (normally 2 weeks, but after the death of a significant other at least 2 months) was skipped, which was criticized as the medicalization and pathologization of the normal human experience of grief. Diagnoses like binge eating disorder, mild cognitive disorder, and disruptive mood regulation disorder in childhood were introduced. These decisions were heavily criticized, most prominently by the psychiatrist who led the development of DMS-IV, Allen Frances. In fact, Frances had been arguing for years that DSM-5 was on the wrong track by introducing more and more disorders without taking into account that these will be overdiagnosed in practice and will create millions of new patients as well as justification for medication that is not indicated. In concert with the practices that advertisement for medication in the U.S. is allowed (not in most European states) this would lead to severe individual and societal side effects of overmedication, so the prediction of Frances and many other critics. Notably, he did not shy away to accuse himself of having performed similar mistakes by introducing three diagnoses in DSM-IV which he now regards as a mistake: attention deficit hyperactivity disorder (ADHD), child bipolar disorder and the Asperger-syndrome (a form of high-functioning autism). In his book “Saving Normal” (Frances, 2013) he argues that DSM-IV has been and DSM-5 will even be more leading to overdiagnoses, to pathologizing normal children and to the treatment of only slightly dysfunctional persons at the expense of taking care of the severely ill.

Here I will not discuss his arguments and the truth of his prognosis in detail, although it is highly likely that some of his predictions will become true, but rather point to an event surrounding the introduction of DSM-5 that makes the claims of the third wave of biological psychiatry clearer.

## THOMAS INSEL’S ATTACK ON DSM-5

The date of the launch of DSM-5 at the APA meeting on May 18th was long known to everybody in the field. So it was probably not by pure chance that just 3 weeks earlier, on April 29th a blog was posted by Thomas Insel, a renowned neuroscientist himself (working in particular on oxytocin and vasopressin in animal research) and since 2002 director of the National Institute of Mental Health, the world’s largest research institute investigating psychiatric disorders. He declared that “the weakness (of DSM-5) is its lack of validity. Unlike our definitions of ischemic heart disease, lymphoma, or AIDS, the DSM diagnoses are based on a consensus about clusters of clinical symptoms, not any objective laboratory measure. In the rest of medicine, this would be equivalent to creating diagnostic systems based on the nature of chest pain or the quality of fever. Indeed, symptom-based diagnosis, once common in other areas of medicine, has been largely replaced in the past half century as we have understood that symptoms alone rarely indicate the best choice of treatment” (Insel, 2013). This is a harsh judgment. And he also drew consequences: “That is why National Institute of Mental Health (NIMH) will be re-orienting its research away from DSM categories.” This is quite a severe conclusion: just before the official diagnostic textbook of the APA is published after more than a decade of work, the largest research organization on mental health declares that it will orient its research away from DSM categories. Why? “(I)t is critical to realize that we cannot succeed if we use DSM categories as the “gold standard.” The diagnostic system has to be based on the emerging research data, not on the current symptom-based categories. Imagine deciding that ECGs (=electrocardiograms, H.W.) were not useful because many patients with chest pain did not have ECG changes. That is what we have been doing for decades when we reject a biomarker because it does not detect a DSM category. We need to begin collecting the genetic, imaging, physiologic, and cognitive data to see how all the data – not just the symptoms – cluster and how these clusters relate to treatment response” (Insel, 2013).

So, in a nutshell: psychiatry has not been able to develop any objective laboratory test for clinical use because the current development of such tests is based on superficial criteria (symptoms), but not on the causal explanatory structures that underly them. If these structures exist he is right: it is difficult to make progress if you are measured by the fit with a descriptive, possibly faulty diagnostic system.

But there are further, homemade, problems within scientific psychiatry. Shitij Kapur, the Dean of the Institute of Psychiatry in London, and coauthors, among them Thomas Insel, gave three possible explanations for slow progress (Kapur et al., 2012). First, many studies in biological psychiatry are underpowered, i.e., they perform p-value chasing with small numbers of subjects (or animals). A good example is psychiatric genetics, but the same argument has been put forward for neuroscience in general (Button et al., 2012). Secondly, many studies are only approximately replicated, i.e., with different methods, different scanners, different paradigms, making it difficult to judge whether an effect is really stable. Thirdly: many stable effects, i.e., effects with large effect sizes are only found in extreme comparisons, i.e., by comparing patients with healthy controls. However, for clinical purposes it would be much more interesting to compare different patient populations. Kapur et al. (2012) also suggest methods to improve the situation, including to increase power, share data, and to report data more accurately. Most importantly, they argue for a stratified medicine (and psychiatry), i.e., for the identification of biomarkers or cognitive tests that stratify a broad-illness phenotype into a finite number of treatment-relevant subgroups. To put it into their metaphor of jacket producing: not to hope for a jacket with one-fits all (the usual approach) but also not hoping for a personally tailored jacket (like in the overambitious project of personalized medicine) but rather to go for a series of chest sizes of jackets for different groups.

## RESEARCH DOMAIN CRITERIA: COGNITIVE SYSTEMS, NEURAL CIRCUITS, AND DIMENSIONS OF BEHAVIOR

A paradigmatic example of how the third wave of biological psychiatry is trying to get a grip on mental disorder and their underlying mechanisms is the initiative of research domain criteria (RDoC) developed by the NIMH which has been suggested as an alternative to investigate mental disorders and develop new classifications that are based on observable behavior and neurobiological measures. According to Morris and Cuthbert (2012) it developed out of two initiatives that targeted schizophrenia, in particular the MATRICS (measurement and treatment research to improve cognition in schizophrenia) and the CNTRICS (cognitive neuroscience treatment research to improve cognition in schizophrenia). RDoC can be regarded as a generalization of these initiatives being constructed for application to all mental disorders. It is based on three central assumptions: (1) mental disorders are presumed to be disorders of brain circuits. (2) Tools of neuroscience, including neuroimaging, electrophysiology and new methods for measuring neural connections can be used to identify dysfunctions of neural circuits. (3) Data from genetics research and clinical neuroscience will yield biosignatures that will augment clinical signs and symptoms for the purposes of clinical intervention and management. It also includes developmental processes and interaction with the environment as orthogonal dimensions that should inform hypotheses and conclusions derived from the RDoC organization structure. This structure is organized as a 2-dimensional schema. One dimension includes constructs that represent five core domains of mental functioning: Negative valence systems, positive valence systems, cognitive systems, systems for social processes and attention/arousal systems. Each of these domains includes subconstructs (around five). For example the negative valence systems include: active threat (“fear”), potential threat (“anxiety”), sustained threat, loss and frustrative non-reward. To take another example: the cognitive systems domain comprises attention, perception, working memory, declarative memory, language behavior, and cognitive (effortful) control. The second dimension consists of units of levels of organization on which the constructs can be measured. These levels are defined as follows: genes, molecules, cells, circuits, physiology, behavioral, self-reports, and paradigms. The “circuits” unit of analysis refers to measures that can index the activity of neural circuits, either through functional neuroimaging or through recordings previously validated as circuit indices (e.g., fear-potentiated startle). “Physiology” refers to well-established measures that have been validated by assessing various constructs, but that do not measure brain circuit activity directly (e.g., heart rate, cortisol). “Behavior” may refer either to systematically observed behavior or to performance on a behavior task such a working memory. The advantage of this conceptualization in comparison to a purely symptom and course based system like DSM is that it is based on research on different levels, allows to characterize patients dimensionally, not categorically (diagnosis present or not) and that it is open to new evidence. Clearly, it cannot simply substitute DSM, which is based on long clinical experience, but it will inform classification based on multilevel science and might, in the long term, identify subgroups of patients that show characteristic constellations within this matrix that are helpful for categorization, treatment or management of patients. In the above mentioned blog Thomas Insel has announced that the NIMH will try to fund studies which follow such a transdiagnostic, systematic approach instead of studies that try to find neural correlates of categories that are simply based on the (superficial) clustering of signs and symptoms.

## EVALUATING THE THIRD WAVE OF BIOLOGICAL PSYCHIATRY: A VIEW FROM INSIDE

By now the general approach or framework of the third wave of biological psychiatry should have become clear. It is focusing on a research-inspired, multi-level approach to understand what psychiatric disorders are, what mechanisms underly signs and symptoms and how an understanding of those mechanisms might help in classification, diagnosis, prognosis, and treatment. Note, that the approach does not entail the claim that biological approaches in a narrow sense are the best therapeutic approaches. It is as such neutral to the question what intervention will prove best to treat whatever there is. For example it may very well be that psychotherapeutic approaches will emerge as the best way to treat certain types of disorders. In fact, psychotherapists see no general problem in integrating their approach into such a framework as psychological mechanisms and principles that are effective in psychotherapy can be conceptualized as part of cognitive neuroscience itself (Walter et al., 2009; Disner et al., 2011). Also, the role of psychosocial and cultural factors can be integrated effortlessly as the MPC approach by Kendler et al. (2011a),b makes clear: if social factors or societal and cultural mechanisms are part of the causal machinery that contributes to the instantiation of typical clusters of signs and symptoms that characterize psychiatric disorders they are part of the underlying explanatory structure.

However, probably many or at least some people will still view this approach skeptically. Indeed, there are several problems and limitations. To name just four of them: first, it could still be argued that the framework favors the neurobiological over other factors, as it entails the idea that psychiatric disorders are brain disorders. It will make no difference if you call psychiatric disorders “disorders of the brain” or “disorders of brain circuits” and thus do not justice to the mental within the concept of mental disorders. Second, the third wave does not include a solution to the normativity problem, namely the question when a constellation of psychological signs and symptoms is already a disorder or when it is still part of “normal experience,” so it will still promote a medicalization of life problems. Third, even if we somehow could solve the first two problems, it might be argued that a focus on the brain will lead to inefficient resource allocation because the outcome for patients is not worth the effort be put in. History has shown that all general claims that we will in the near future know “the” causes of mental disorders have failed, and the continuous failure of neurobiology (with some exceptions) to sufficiently explain or predict mental disorders shows that it cannot account for such complex phenomena. Therefore, we should rather focus on the well-known psychosocial factors contributing to the development or sustainment of psychiatric disorders which are much more relevant in practice.

A recent critique of the thesis that “addiction is a brain disease” can be interpreted as a condensed combination of these worries. It argues that addiction would only be a brain disease if it has (i) neural correlates, (ii) these correlates are pathological and (iii) that pathology is sufficient for the person to have a disease, in almost any accessible environment Levy, 2013. As addicts are able to quit in certain environments, addiction would not qualify as a brain disease. This is a very clever argument as it uses one feature of the multilevel approach, namely the role of environmental factors, to argue against the “disorder of brain circuits thesis.” Indeed, there is a grain of truth in this argument, but only insofar as it helps to distinguish “organic or neurological” from “mental or psychiatric” disorders. For example, neurodegenerative diseases like M. Huntington or Alzheimer will progress in almost any environment, whereas drinking might stop. However, there are two problems with this argument: first of all, it confuses behavior (drinking) with the disorder (alcohol addiction). It is well known that people suffering from alcohol addiction who manage to quit, still are addicted life-long and have a high propensity for relapse – exactly this might be explained by the brain disorder thesis. Secondly, the argument puts the stake much too high. Using the same kind of argument it could be argued that phenylketonuria, a genetically transmitted severe metabolic disorder is not *really* a metabolic disorder as it can be effectively treated by a diet, i.e., the pathology is not sufficient for a person to have a disease in almost any accessible environment.

Finally, some may argue, that also the third wave of biological psychiatry, like the preceding waves, will tend to devalue an approach to psychiatry that focues on the personal level. For example, the concept of MPC is based on the idea that regards minds as brains and brains as kind of machines that are causally effected by different levels. This approach, so the argument may go, ignores the personal level even if it may pay lip service to the subjective by for example including “subjective reports” in the RDoC grid.

There are several ways to response to these critiques from within, some of which I will mention here. First, admittedly, there is a common misunderstanding on the role of neurobiological findings in psychiatric disorders. Very often, it is either said, implicitly assumed, or implied that the mere fact that there is a neurobiological correlate of a mental dysfunction is already a proof that the “causes” of the respective disorder are biological in the same way as for neurological disorders. But this clearly is a misconception. Because every mental state has a correlate in the brain, we should be able to find at least in principle neurobiological correlates of any mental state, pathological or not. So the question is not, whether there is a neurocognitive correlate or mechanism, but whether it is pathological, how it came into being, whether it is persistent, whether and how it can be influenced, and so forth. In fact, the neurobiological misunderstanding even goes further in many cases as often it is wrongly concluded that the existence of a “brain signature” (to use a more neutral term) would already imply that the disorder cannot be controlled or changed by psychological means, or even that it is inborn or genetically caused, implications which clearly are non-sequitures, but widely believed.

Second, the normative problem indeed has to be addressed – not only by biological psychiatry, but also by any other approach to psychiatry, and not only for psychiatric but also for all concepts of disorders – and consequently it has been discussed in medicine in general. As it is in no way specific for psychiatry, let alone biological psychiatry, I will not discuss it here in detail but just make some remarks. It is clear that the sheer discovery of neural correlates or mechanisms of a disorder cannot prove a state as pathologically. This can be done only by spelling out a concept of normal functioning. If a biological approach claims to be able to define mental disorders without reference to norms it must fail. Normativity in the context of mental disorders comes at least in three guises, “statistical,” “biological design” or “value-preference laden” (Graham, 2013, p. 59). For example, most definitions of mental disorders include a criterion of suffering or of clinical relevance, that only can be spelled out with respect to a norm that cannot be read simply from biological facts. I will return to this issue later. Although it has to be admitted that the third wave of biological psychiatry does not take a specific stance to the normativity problem, it should be noted that this can be only used as a critique against variants of biological psychiatry that explicitly claim that normality can be inferred simply from biological measures.

Thirdly, why has neurobiology failed to deliver better results for explanation, diagnosis, prognosis or treatment? Some answers relating to methodological problems have been already discussed above (Kapur et al., 2012). However, a further explanation for only modest progress is often not mentioned. These are the ethical constraints under which biological psychiatric research has to operate which does make progress difficult. In contrast to other medical disciplines psychiatric research can access the “organ of the mind,” the brain, only indirectly. There is no known ethically justifiable way to directly access brain tissue to investigate assumed molecular mechanisms. In contrast, the heart, the liver, the kidney and many other organs can be accessed directly in therapy and research by taking biopsies or measuring metabolites in the blood. There are only a few exceptions to this barrier, for example the possibility to measure certain molecules non-invasively with magnetic resonance spectroscopy, or with research windows related to invasive therapeutic procedures in epilepsy surgery or deep brain stimulation. Direct access to the brain in animal research also has its problems, because rodents and humans differ in many respects and animal experiments are confronted with ethical issues, too. So the “failure” of biological psychiatry is not necessarily related to its concepts or theoretical approach, but partly may be explained by important and relevant ethical barriers we have implemented in human research for good reasons.

Fourthly, does a biological psychiatry approach imply disrespect for persons? First note, that this critic in its most general and radical form is not confined to biological psychiatry but to any psychiatric approach that claims that there are mental disorders in the first place. This antipsychiatric argument claims that mental illness in general is a myth by confusing sickness with life difficulties and by stigmatizing people with mental problems as having a disorder and thus not giving them the credit and responsibility for what they do and chose to be. In a more specific and much less radical, but more frequent variant (not claiming the non-reality of mental disorders) a biological approach of psychiatry is accused of resulting in an overenthusiastic reliance on medication and an insufficient use of understanding the life stories and real-world problems of patients. Without doubt, overmedication is a problem in certain strands of psychiatry and admittedly this may be due to the fact of an oversimplified picture of mental disorders (“For depression you need to substitute serotonine like insuline in diabetes”). However, many of these implications are not inherent to the concepts of the third wave of biological psychiatry but rather are based on older conceptions that postulated a close connection between etiology and therapy, that has been abandoned today in current practice. For depression for example there was a distinction between endogenous depression (from within, medication, no talking cure), neurotic depression (originating in childhood, talking cure, no medication) and reactive depression (understandable reaction after a life event).

The aforementioned responses to a critique to biological psychiatry were given from within psychiatry and psychiatric research in itself. Many of these issues revolve about the “disorder” part of mental disorders. However, I think that a more comprehensive way of assessing the prospects of biological psychiatry can only be found when we turn to the “mental” part of a theory of mental disorders. In order to do so we can turn to a rich resource that has reflected on the concept of the mental for a long time: philosophy of mind.

## RECONSIDERING BIOLOGICAL PSYCHIATRY: A PHILOSOPHY OF MIND PERSPECTIVE

If we want to understand what mental disorders are then we should take the question what “the mental” is more seriously. Traditionally, there has been a close link between philosophy in general and psychiatric theorizing. Here, I will restrict myself to recent philosophy of mind approaches, as they are targeting similar problems as biological psychiatry: what is the connection between mind and brain? The idea behind consulting philosophy is simple: if we better understand how mental states are related to brain states we might better understand how disordered mental states relate to disordered brain states. Take for example the thesis of identity theory that assumes that mental states are identical with brain states. If this is true, it seems to follow straightforwardly that disordered mental states simply are disordered brain states. Or take the problem of reductionism and mental causation: if we were really able to show that mental states can be reduced to brain states, this would leave us with only two possibilities: either we have to eliminate mental states, because they are nothing more than a convenient, folk psychological way to talk about hidden brain states or we have to conclude that mental states are epiphenomenal, i.e., have no causal powers. This seems like a conclusion only few people would like to embrace. Or take the idea of dualism. Do we have to assume a special substance that does all the work in explaining mentality that is in a separate ontological realm outside of physical reality?

However, if we dwell too deep into the heart of philosophy of mind, the danger is great, that we will end up with metaphysical debates that might too easily be dismissed as theoretical talk with no direct relevance for psychiatry. Instead, I will refer here to two examples of the relevance of philosophy of mind for psychiatry: one specific approach of a theory of mental disorder by a philosopher (George Graham) and one family of problems discussed in contemporary philosophy of mind, namely if mental states extend beyond the brain in a relevant sense.

A comprehensive and accessible version of linking philosophy of mind and mental disorders has been given by Graham (2013). In his theory he explains what mental disorders are, according to which (normative) criteria we classify them as clinically relevant and how they differ as mental disorders from proper brain (=neurological) disorders. According to Graham a mental disorder is a disability, incapacity or impairment in one or more basic or fundamental mental faculties of psychological capacities of a person that has harmful or likely harmful consequences for its subject. It is a *disorder* because it is harmful and undesirable for the subject, whether the subject himself appreciates this or not. In more concrete terms this means that the person is worse off with than without the disorder, that the disorder has a non-voluntary and personally uncontrollable nature and that the disorder cannot be excised or extirpated by the mere addition of other psychological resources. For example, the delusion of a paranoid person will not be alleviated by giving more information about the content of his delusion and the sadness of a depressed person will not be cured by cheering him up. Mental disorders are *mental* disorders because they are brought about by a mix of mental forces and brute a-rational neural mechanisms, or at least Graham argues so. The crucial point here naturally is what Graham means by mental forces. He explicitly states that he is not a dualist. Rather, he tries to argue what the “mental” in mental disorders refers to. The mark of the mental is that states of mind are constituted by either or both of two elements, i.e., consciousness and intentionality. Only if the causal mechanisms bringing about or sustaining a mental disorder work through conscious and/or intentional states, so Graham claims, they should be categorized as mental disorders. Mental symptoms that arise from brute brain affections (like stroke, neurodegeneration, or infection) are neurological disorders even if they present with (secondary) mental symptoms. Also, the mental is decisive for the criteria when a mental state of mind should be regarded as a disorder and not as a variant of normal mind life: namely when they impact a person’s reason-responsiveness or rationality considerably without totally destroying it.

In Graham’s theory the mental plays a prominent role in several respects: first, because the mechanisms causing or sustaining mental disorders are supposed to work through those brain mechanisms that implement mental (intentional and/or conscious) states and thus through mental qua mental. Second, the normative criteria for clinical relevance (and thereby the criteria for separating normality from disorder) rely on the impairment of intentionality and rationality, i.e., marks of the mental. Thirdly, he argues that mental disorders (like panic attacks, schizophrenia, depression) should and can be distinguished from proper neurological brain disorders (like stroke, Parkinson, Alzheimer) by the fact that the latter are brought about by pure mechanical, brute, a-rational affections of the brain that moreover are not sensitive to psychological (mental) treatment. In contrast, the “mental” in mental disorders has a double role: first it is characterized by an impairment of intentionality and rationality and second, because these marks of the mental are not totally absent but within the symptoms there is still a sense of rationality and intentionality preserved.

A problem in Graham’s theory is his explication of mental forces. Sometimes, he seems to imply that rationality or intentionality have a causal power of their own, although he denies that. But the worth of his approach for biological psychiatry seems for me that he insists on the relevance of the role of the mental in understanding, explaining and identifying mental disorders against pure brain disorders and non-pathological mind states on the other. In fact, many proponents of biological psychiatry now accept an interplay of neurobiological and psychological (mental) factors. However, if the mental is identical with the neural what does this claim of interaction amounts to? So let us turn then to the important question, if the mental can really be reduced to the neural.

In philosophy and in cognitive sciences there exist a number of proposals that doubt that cognitive processes (for our purpose: mental states) are best understood as only internal processes that happen within a cognitive system (in our case: the brain). Internal approaches, so the basic idea, ignore that cognitive processes are *situated*, i.e., that they essentially depend on (weak version), or even may be constituted by (strong version), their embodiment and the interaction with the natural, technological and social environment. There is not yet a consistent or complete theory of situatedness, rather there are several strands of research and theorizing that can be subsumed under the catchword “the 4Es”: the embodied, extended, embedded and enacted mind (Lyre andWalter, 2013). The main idea is that in order to understand what cognition (the mental) is, it is necessary to take into account that cognitive capacities of a system may depend on the fact that those systems (our brains) are (i) *embodied*, i.e., coupled to our bodily constitution and that it therefore is necessary to regard the bodily realization of cognitive abilities as an integral part of the cognitive architecture; (ii) situationally *embedded*, i.e. are dependent in a specific way on their environment, i.e., cognitive systems exploit the specific circumstances of their environmental context in order to increase their performative abilities, (iii) *extended*, i.e., extend over the boundaries of our body into the technological or social environment and thus are constituted not only by internal factors but also by external, environmental factors and (iv) *enacted*, i.e., arise only by the active interaction of an autonomous systems with its environment (Walter, 2010).

The thesis of embodiment has a long tradition in phenomenological philosophy, e.g., in the writings of Merleau-Ponty. The thesis of the extended mind has more recently been introduced into the debate by a paper published in 1998 (Clark and Chalmers, 1998). They introduce an example of an external device for memory (a cognitive process) that has since then been discussed extensively in the literature. The example refers to the notebook of Otto, an Alzheimer patient with memory problems who uses his notebook instead of his normal physiological memory in order to remember certain things. The argument is that if the entries into the notebook play the same role in Ottos life and in the explanation of his behavior as neurally implemented memory contents in healthy adults, it would be arbitrary or neural chauvinism if we would not regard them in the same way as genuine parts of the material substrates of his normal memories and beliefs. The general form of the argument inherent in this example is called the *parity principle*: if a part of the world functions in a way that, would it happen in our brain, we would have no hesitation in recognizing as part of a cognitive process, then that part of the world is part of the cognitive process. To make this part of the process more plausible it is easy to modify the example such that the notebook is constructed as a brain-computer-interface, e.g., as a digital device coupled more directly to the brain, for example in a technological advanced form of the actually existing google glasses.

Why could the 4E thesis be relevant to understand the nature of mental disorders? Because they regard processes external to the brain as constitutive for mental processes and thus also as constitutive for disordered, pathological mental processes. An example, where this might be relevant is ADHD. ADHD might be only correctly diagnosed as a mental disorder if the external world is such that adolescents grow up in an environment that favors attentional distraction and punishes hyperactivity. In a similar vein, anorexia nervosa, a severe and often deadly mental disorder in Western countries seems to be much less frequent or even non-existent in environments in which a slim figure and control of eating and weight is not promoted, like in poor countries in Africa. These facts seem to draw into doubt that every currently acknowledged mental disorder is best categorized as a pure brain disorder – which is not to deny that internal processes of the brain play an important role if specific circumstances hold.

The main point which I would like to make here is that biological psychiatry has to take into account theories about how the mental is constituted. The new wave of biological psychiatry might be able to incorporate these issues into its conceptualization of mental disorders – but only if it comes along with a consistent theory of the mental that should take into account arguments and insights of philosophy of mind.

## Conflict of Interest Statement

The author declares that the research was conducted in the absence of any commercial or financial relationships that could be construed as a potential conflict of interest.
